# Associations between macular pigment, iris color and reflectance, ethnicity, and color vision: An observational study

**DOI:** 10.1371/journal.pone.0220940

**Published:** 2019-08-08

**Authors:** Roya Garakani, Jason S. Ng

**Affiliations:** Southern California College of Optometry, Marshall B. Ketchum University, Fullerton, CA, United States of America; Save Sight Institute, AUSTRALIA

## Abstract

**Purpose:**

Conflicting findings exist in the literature with regard to the relationship between iris color, ethnicity, macular pigment optical density (MPOD), and hue discrimination. This study re-examined these relationships, accounting for factors that may have confounded prior studies. Clinically, the relationship between MPOD and hue discrimination may impact the utility of macular pigment supplementation as a treatment for conditions such as macular degeneration.

**Methods:**

Subjects (n = 30, mean/SD age = 25.1/2.5 yrs.) with normal color vision completed MPOD testing and Farnsworth-Munsell 100 hue (FM100) testing. MPOD data was derived from the average of three measurements using the QuantifEYE II device and FM100 testing included training runs. The total error score of the FM100 test was used for analysis. Iris color was determined subjectively, while iris reflectance was derived using calibrated iris images. Spearman correlations were used to determine the relationship between MPOD and FM100 test scores. Kruskal-Wallis testing was used to investigate MPOD differences among different ethnicities and iris colors.

**Results:**

MPODs were normally distributed with a mean/SD = 0.38/0.13. Total error scores had a mean/SD of 10.7/9.7, but were not normally distributed. Iris reflectances had a mean/SD = 11.0/8.7. MPODs were not correlated to total error scores (p = 0.93). MPODs were also not correlated with iris reflectances (p = 0.28) even though MPODs differed significantly by iris color (brown = 0.44, hazel = 0.31, blue = 0.33, p = 0.04). Iris reflectances were not correlated with total error scores (p = 0.68). MPODs differed significantly (p = 0.003) between Asian and Caucasian subjects, 0.44 and 0.33, respectively.

**Conclusions:**

This study did not find a correlation between MPOD and hue discrimination as in some previous studies. While MPOD was associated with iris color and ethnicity as found in prior studies, it was not associated with iris reflectance, which may be a better indicator of ocular pigmentation compared to either iris color or ethnicity.

## Introduction

The associations between hue discrimination (i.e. color vision), iris color, ethnicity, and macular pigment optical density (MPOD), are unclear in the literature. Hue discrimination is commonly measured by a clinical test known as the Farnsworth-Munsell 100 Hue (FM100) test. This test includes areas where patients with either red-green or blue-yellow defects may make errors. Studies have found that scores on the FM100 test can be affected by various factors, such as illumination level, practice, age, pupil size, ethnicity, iris color, and MPOD.[1–10]

Older age contributes to higher (i.e. worse) FM100 scores because retinal illuminance declines with age (due to pupil miosis and increasing crystalline lens density) and color vision declines rapidly at lower light levels.[3] Small pupil sizes, at any age, would also be expected to result in higher FM100 scores due to less retinal illuminance.[4] While an association between ethnicity and FM100 scores has been shown in the past, it was thought that ethnicity was merely acting as a surrogate measure of MPOD levels.[5] Similarly, iris color as a surrogate measure of MPOD has been associated with FM100 performance.[4] In fact, darker iris coloration has been found to be associated with higher MPOD levels on average.[6, 7] A previous study by Murray et al. demonstrated a consistently higher MPOD with dark irises compared to light ones using the same MPOD device used in our study.[2]

Macular pigment has a peak absorption at 460 nm[11] and acts as a yellow filter that causes tritan-like (i.e. blue-yellow) defects in color normal patients[12] A simulation study showed that a higher MPOD caused higher FM100 scores,[13] and clinical studies have attributed higher FM100 scores to higher MPOD levels.[4, 5] However, another study did not find any correlation between FM100 scores and MPOD levels.[10]

Investigating the relationship between FM100 scores and MPOD levels may help partially elucidate why there is such a wide range of hue discrimination seen in color normal patients, as shown by normative data for the FM100 Hue test.[14, 15]The cause for this wide range (i.e., a large amount of variability in the color normal population) has not been thoroughly investigated. While age and distribution of S, M, and L cones can play a role, these factors alone would contribute to a normal distribution in the range of FM100 scores in color normals, which has not been demonstrated.[3, 16, 17] If MPOD levels are directly associated with hue discrimination, this could potentially be an undesired side effect of MPOD supplementation that patients and providers should consider. Lower levels of MPOD have been shown to be a risk factor for age-related macular degeneration as well [18, 19] and supplementation of MPOD has been shown to improve retinal function [20] as well as visual performance.[21, 22]

Darker iris color and Asian ethnicity have been shown to have a correlation with higher MPOD.[2, 4–6] As coloration is somewhat a surrogate measure of the level of pigmentation of the anterior iris, this study used iris reflectance, rather than coloration. Using iris reflectance provides a more objective and finer grading scale outcome measure than coloration alone and thus may allow a better metric to investigate any associations with MPOD levels. If iris reflectance is correlated with MPOD, it could be used as a surrogate measure when MPOD measurements are not possible.

We hypothesized that higher MPOD values would be associated with poorer blue-yellow hue discrimination with FM100 testing. We also hypothesized that darker iris color and lower iris reflectance would be associated with higher MPOD values. This study sought out to test these hypotheses and control for the possible confounding factor of FM100 learning, which none of the aforementioned studies addressed. We also re-examined the relationships between hue discrimination, MPOD, ethnicity, and iris color.

## Materials and methods

### Subjects

Marshall B. Ketchum University IRB approved the study and assigned the approval number “14-t1”. Written consent was obtained from all subjects. Thirty subjects (13 males, 17 females) were enrolled in the study and had a mean ± SD age of 25.1 ± 2.5 years. Self-reported iris color at the time of enrollment was fairly evenly distributed (i.e. brown = 10, hazel = 9, blue = 11). There were no requirements as to subject race, gender, or occupation. Despite this, nine Asian subjects and twenty-one Caucasian subjects enrolled in the study, allowing examination of ethnicity in the data analysis. Subjects had to have at least 20/25 visual acuity and be between 18 and 40 years of age. They could not have any inherited or acquired color vision deficiency or any ocular pathology. While complete eye examinations were not part of this study, every subject reported having had a comprehensive eye examination within the past 12 months at our University clinic. All subjects provided written informed consent. The procedures complied with the Declaration of Helsinki and were reviewed by the University institutional review board.

### Sample size

In order to calculate sample size, the primary outcome measure chosen *a priori* was the square root of the total error score from the FM100 test. The square root of the total error score has been shown to be normally distributed in many prior studies. Based on prior literature, the presumed ranges of the square root of the total error score and the MPOD values were 0 to 15 and 0–1.4, respectively. The presumed means and standard deviations (mean ± SD) of the square root of the total error score and MPOD values were 7.0 ± 2.5 and 0.40 ± 0.16. The FM100 values were based on data from Verriest et al, (age: 20–29, n = 29, mean ± SD = 5.69 ± 2.07) and Mahon and Vingrys (n = 126, mean ± SD = 6.12 ± 2.02).[14, 16] The MPOD values were based on data from Bartlett et al. (mean ± SD = 0.35 ± 0.14) and van der Veen et al. (mean ± SD = 0.33 ± 0.19).[23, 24] Given these expected values, in order to detect a significant Pearson correlation coefficient of 0.50 with a statistical power of 0.80 (*β* = 0.20) and an alpha level of 0.05, the expected sample size needed was determined to be 25 subjects. We recruited 30 subjects to be conservative.

### Overall materials and procedures

All subjects completed a case history, visual acuity testing, color vision testing (i.e. Richmond 4^th^ edition HRR color test screening, FM100, and anomaloscopy), MPOD testing, and had anterior segment images to measure pupil size and iris reflectance. Only one eye of each subject was used for the final data analysis and that eye was identified by MPOD testing.

### MPOD testing

MPOD levels were determined using the QuantifEYE II, also known as the MPS (Elektron Eye Technology, Cambridge, UK), which is a validated device that uses heterochromatic flicker photometry.[23, 24] The device takes foveal and peripheral measurements to provide a psychophysically derived MPOD value. The primarily stimulus is a 1-degree spot that flickers between blue and green. When the flicker is above the critical flicker frequency, the subject perceives a static cyan target. The flicker rate is decreased until the subject detects the flicker. This is repeated for varying blue/green intensity ratios. A reliability report of good, borderline, or poor was provided along with the MPOD value. All MPOD values analyzed in the study had a reliability of ‘good’. MPOD measurements for both eyes were then taken and it was decided *a priori* that the eye with the higher MPOD would be used for the study (and all other testing would include only that eye as well) in order to include the highest MPOD levels in the study analysis. Once the eye with the highest MPOD was identified, the MPOD for that eye was tested two more times and the average of the three measurements was used for data analysis.

### Anomaloscope testing

Rayleigh (red-green) matching ranges (and midpoints) and Moreland (blue-green) matching points (an average of 6) were determined for all subjects on an anomaloscope (Oculus HMC, Germany) using neutral adaptation.

### FM100 testing

A new FM100 test (X-Rite, Grand Rapids, Michigan) was for this study. The standard test distance of 50 cm was used for the FM100 test. At this distance, each cap from the test subtends 2.0 degrees at the eye, which is within the spatial distribution of macular pigment.[25] To account for the learning effect that had previously been proven to occur with the FM100 test, the subjects completed a training procedure before the final FM100 test was conducted. The training procedure was taken from Breton et al.’s study[26] and has been shown eliminate the learning effect, making FM100 scores much more repeatable.[26] The training procedure consisted of normal verbal instruction given for the FM100 Hue test, followed by an instructional poster with examples of correct and incorrect cap sequences. The poster emphasized rechecking sequences of 3–4 caps to make sure there were no incorrect placements. A ten-cap subset of the FM100 Hue test (caps 47 to 56) was shown to the subject, and they were required to order them. Feedback was provided to the subject after this trial. The practice trial ended when the subject ordered the ten caps correctly two times in a row, or completed three trials with the range of error scores being four or less, or completed six trials (no matter the score). The majority of the subjects ordered the ten caps correctly two times in a row. Afterwards, as part of the practice trial, a complete FM100 Hue test was administered.

Once the training procedure was completed, the FM100 Hue test was administered. A Richmond illuminator (i.e. illuminant C equivalent) provided an illumination of 500 lux. FM100 results were analyzed by the standard scoring approach (vs. Kinnear’s method)[15] using X-Rite FM100 scoring software.[14] Total error score and the square root error score were derived from the scoring software. Partial error scores were derived from the appropriate subset of cap scores.[27] Partial error score 1 (red-green) included errors in caps 13–33 and 55–75, whereas partial error score 2 (blue-yellow) included errors from caps 76–12 and 34–54.

### Iris color, iris reflectance, and pupil size

As the iris is observed by reflectance (i.e. it is not self-luminous and thus not inherently colored), iris reflectance was studied in addition to iris color. The self-reported iris color was confirmed by the examiner at the time of enrollment and from anterior segment images as blue, hazel, or brown. In a previous study,[28] we determined a procedure to convert iris luminance values into iris reflectances. All subjects had digital anterior segment images taken with a Canon CR-1 camera (Canon, Lake Success, New York). Reflectances from a standard gray-scale 15-step target (Edmund Optics, Barrington, NJ) card were derived and used to calibrate the system by also taking an image of the target with the camera and then measuring the luminances of each step target on a calibrated computer monitor. A luminance vs. reflectance graph was generated based on the gray-scale target and the best-fit linear line was found: target (iris) reflectance = 0.868 × target luminance. This formula was used to convert the luminance measurements taken from the superior, inferior, nasal, and temporal regions of the iris (which were then averaged) from the anterior segment images acquired in the study into a single iris reflectance measurement for each subject. The images were viewed on a single computer monitor and sized to the same dimensions with predetermined measuring areas. Luminances were measured with a Minolta LS-110 spot photometer (Konica Minolta, Ramsey, NJ). Pupil sizes were also measured from the acquired images. During the anterior segment image capture, a standard ruler used to measure pupillary distance was placed in contact with the lower eyelid. This allowed standardized scaling of any feature in the image and using this ruler for calibration, horizontal diameter of the pupil was determined.

### Data analysis

Normality of the data was tested by the Kolmogorov–Smirnov test statistic. Correlations were tested with either the Pearson or Spearman rho where appropriate. Kruskal-Wallis testing was conducted to test for associations between MPOD and ethnicities, iris color, and iris reflectance.

## Results

### Summary

Table 1 shows the mean/SD of the variables measured. The MPOD for five subjects was equal between the eyes, and in those instances the eye was chosen randomly. All subjects correctly identified every blue-yellow and red-green screening plate on the Richmond 4^th^ edition HRR test. Pupil size did not vary by ethnicity (Asian = 5.25 ± 0.65, Caucasian = 5.57 ± 1.43, p = 0.41).

**Table 1:** Summary of results.

	Mean	SD	Min	Max
**Age (years)**	25.1	2.5	19.3	30.9
**VA (logMAR)**	0.05	0.06	-0.20	0.10
**MPOD**	0.38	0.13	0.17	0.63
**Total Error Score FM100**	10.7	9.70	0.00	32.0
**Partial Error Score 1 (RG) FM100**	3.20	4.29	0.00	16.0
**Partial Error Score 2 (BY) FM100**	7.47	7.12	0.00	24.0
**Anomaloscope RG Midpoint**	42.6	2.11	39.3	49.0
**Anomaloscope RG Range**	5.95	2.89	1.00	13.0
**Anomaloscope BY Match Point**	59.5	11.4	29.5	80.4
**Iris Reflectance (%)**	11.00	8.70	0.54	26.24
**Pupil Size (mm)**	5.4	1.3	3.0	8.0

VA = visual acuity; MPOD = macular pigment optical density; RG = red-green; BY = blue-yellow

### Normality

**Fig 1:**
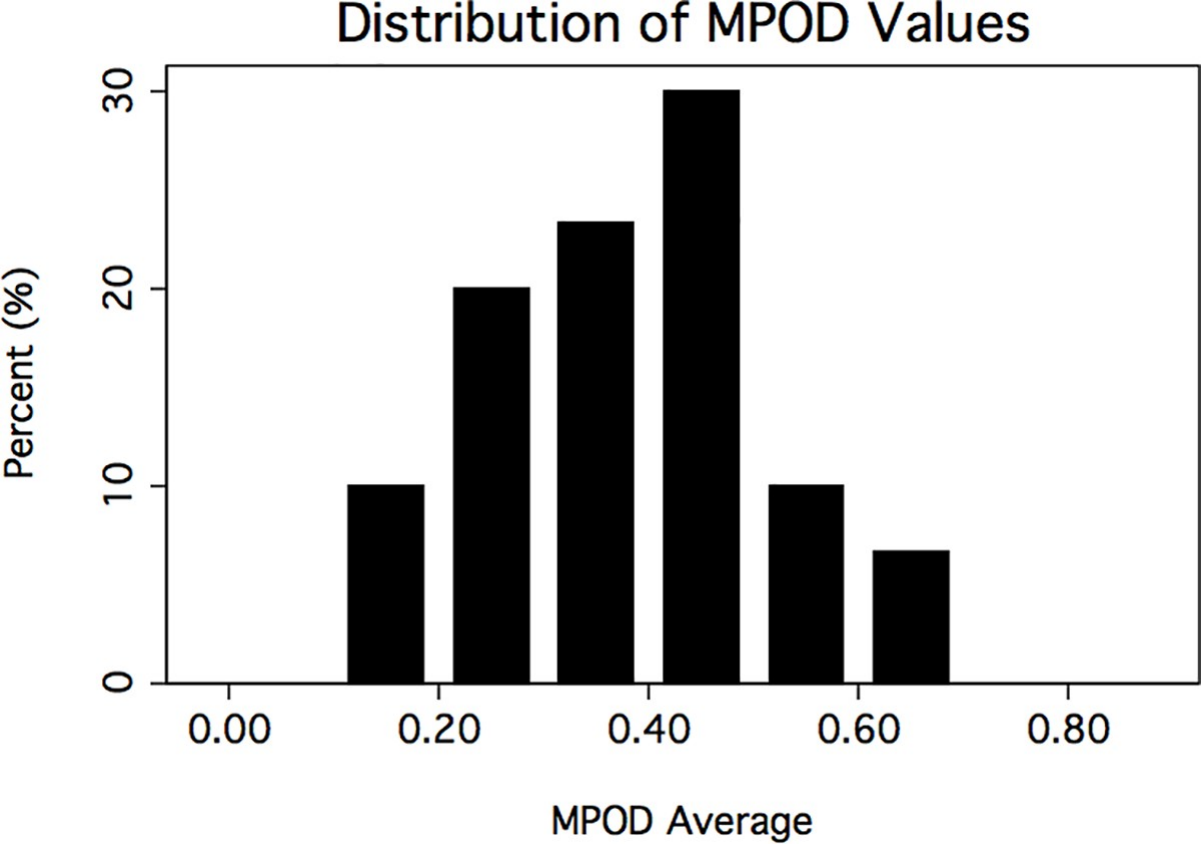
Distribution of the macular pigment optical density (MPOD) average values in the study sample. Each subject had three values averaged.

**Fig 2:**
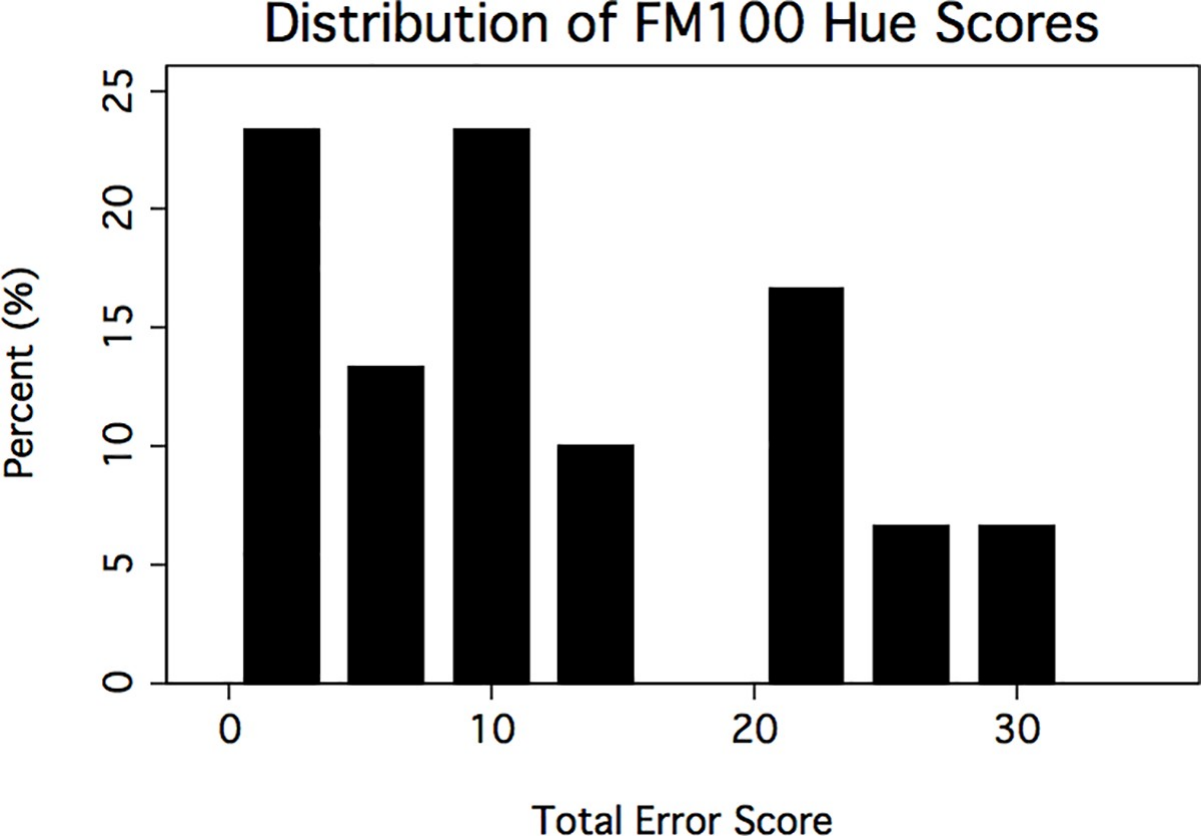
Distribution of Farnsworth-Munsell 100 (FM100) hue total error scores in the study sample.

MPOD results (Fig 1) followed a normal distribution (p = 0.57), but all other results, including FM100 scores (Fig 2), did not follow a normal distribution (p < 0.01). Seven subjects achieved perfect scores on the FM100, which was the apparent cause of non-normal FM100 total error score and square root total error score distributions. Since both the total error scores and the square root of the total error scores were not normally distributed, we chose to analyze total error scores directly as they are more clinically utilized.

### Correlations

**Fig 3:**
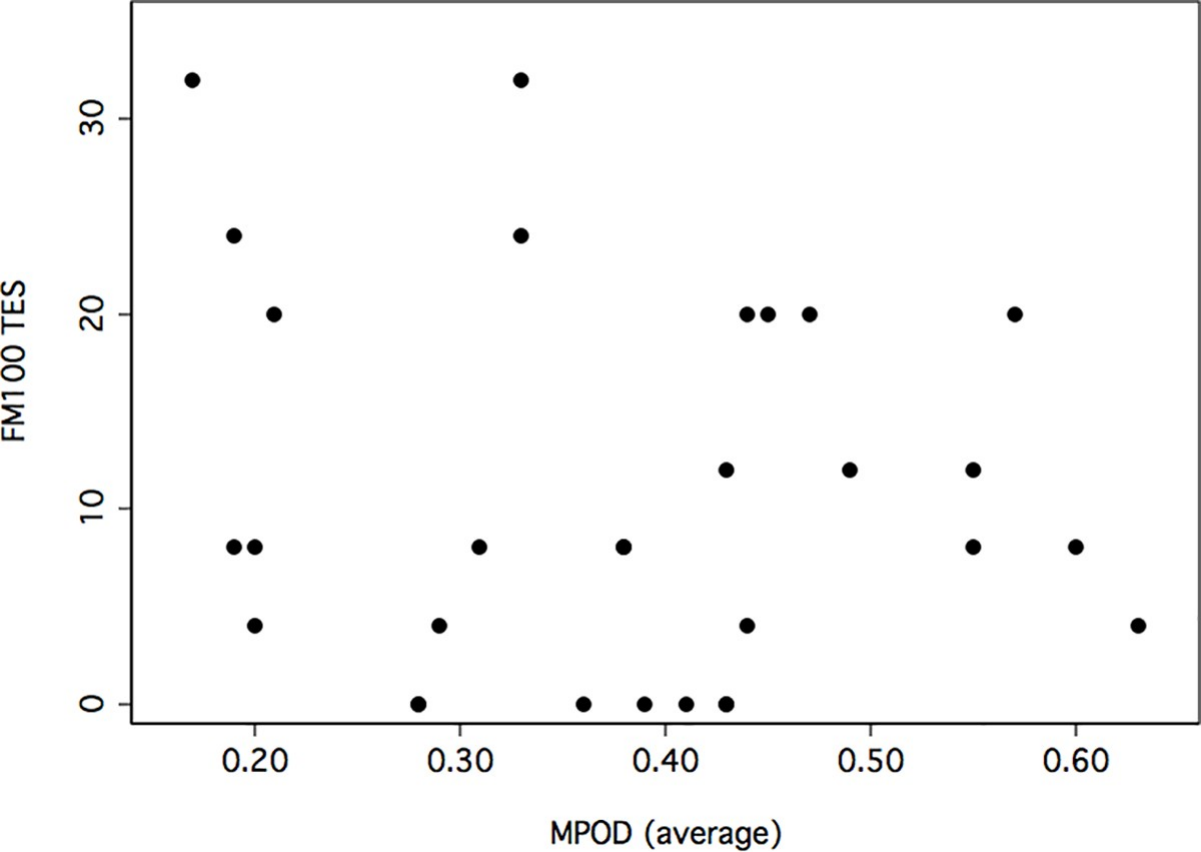
Scatter plot of Farnsworth-Munsell 100 (FM100) hue total error scores (TES) against averaged values of macular pigment optical density (MPOD).

Analysis of correlation between FM100 and MPOD scores was determined by calculating Spearman correlations given the non-normality of FM100 Hue scores. Fig 3 plots FM100 and MPOD scores for each subject. Pearson correlations were also conducted since it related to the sample size planning. The Spearman correlation between total error scores and MPOD average scores was -0.017 (p = 0.93), whereas the Pearson correlation was -0.15 (p > 0.05). Pupil size was not correlated with FM100 scores (p = 0.67). Additional Spearman correlations are shown in Table 2.

**Table 2:** Spearman correlations (top number) and associated p-value (bottom number).

	MPOD	TES	PES1	PES2	RG Range	BY Point	Iris Reflectance	Pupil Size
**MPOD**	-	-0.02 0.93	-0.10 0.59	0.04 0.85	0.02 0.90	0.10 0.59	-0.21 0.28	-0.38 0.04
**TES**		-	0.77 0.00	0.92 0.00	0.46 0.01	-0.19 0.32	-0.08 0.68	0.08 0.67
**PES1**			-	0.48 0.01	0.37 0.04	-0.27 0.15	0.05 0.79	0.03 0.88
**PES2**				-	0.44 0.02	-0.12 0.55	-0.12 0.52	0.07 0.73
**RG Range**					-	-0.01 0.98	-0.06 0.76	0.06 0.76
**BY Point**						-	0.32 0.09	0.16 0.39
**Iris Reflectance**							-	0.18 0.35

MPOD = Macular pigment optical density (average of 3), TES = Farnsworth-Munsell total error score, PES 1 = Farnsworth-Munsell partial error score 1 (red-green), PES 2 = Farnsworth-Munsell partial error score 2 (blue-yellow), RG Range = Anomaloscope Rayleigh matching range, BY Point = Anomaloscope Moreland match points (average of 6).

### Ethnicity and MPOD

Kruskal-Wallis tests showed a significant difference between ethnicities in their MPOD scores. Asian subjects had a significantly higher (p = 0.003) median MPOD score (0.44) as compared to Caucasian subjects (0.33). Asian subject all had brown irides, while only one Caucasian subject was classified as having a brown iris color. Asian subjects had a significantly lower (p = 0.04) iris reflectance (6.67 ± 8.23) compared to Caucasian subjects (12.86 ± 8.40).

### Iris color, iris reflectance, and MPOD

Irides classified as blue, hazel, or brown had mean ± SD iris reflectances of 16.09 ± 8.11, 8.35 ± 7.32, and 7.80 ± 8.54, respectively. Kruskal-Wallis testing showed a borderline significant difference (p = 0.05) among the iris reflectances for the iris colors. Pair-wise Mann-Whitney testing showed that the iris reflectances between blue and brown irides were significantly different (p = 0.035), but were not significant between blue and hazel (p = 0.053) nor between hazel and brown (p = 0.46).

Kruskal-Wallis analysis also showed a significant difference between iris colors in relation to MPOD. Brown-eyed subjects, hazel-eyed, and blue-eyed had mean MPOD scores of 0.44, 0.31, and 0.33, respectively (p = 0.04). However, no correlation was found between iris reflectance and MPOD (p = 0.28).

## Discussion and conclusions

### MPOD

In this study, our subject sample had (mean ± SD) MPOD values of 0.38 **±** 0.13. Using the same instrumentation as this study, van der Veen found very similar MPOD values: 0.40 ± 0.15,[24] indicating a representative subject sample that allows broader generalization of the results found in this study, at least with regard to the distribution of MPOD values in the population.

### MPOD and FM100 hue

There was no significant correlation found between MPOD and FM100 scores. This agrees with the results found by Davison et al.[10], but not with the results found by Dain et al.[4] or Woo et al. [5] The most recent study that attempted to resolve the conflicting results of Woo et al.’s and Dain et al.’s studies was by Davison et al. The researchers used MPOD and FM100 measurements to determine if there was a relationship between the two. Unlike the Woo and Dain studies, the Davison study was the first to measure MPOD, and this present study agrees with Davison et al.’s results. One issue with Davison’s study was that there were no repeated trials for either the FM100 or MPOD measurements, which may be critically important if learning or training effects are possible with the tests. In fact, several studies have shown that FM100 scores improve with practice.[1]

Fine and Kobrick [1] found that individuals show significant improvement in FM100 scores after repeats of the test. On average, total error scores of 36 subjects steadily decreased from 90 to 60 over the course of seven repetitions of the test. Another study found that including a training session for the FM100 Hue test eliminated the learning effect.[26] This resulted in consistent results over five repetitions of the test. Thus, in prior studies FM100 results may have been more variable than necessary, and they in fact may not represent the ‘true’ FM100 scores of the subjects, which would prevent an accurate assessment as to whether any association existed between MPOD and FM100 error scores.

A floor effect was seen in the FM100 score with the subjects that participated in this study likely due to the training, as well as the young average age of our participants and the higher illumination source used. The relatively high number of subjects that had perfect scores created a floor effect that has not been seen in other studies, like Dain et al.’s and Woo et al.’s. Dain et al.’s study found a square root total error score of 5.29 in the 32 females in their study, and a square root of the total error score of 4.32 in the 30 male subjects in their study. Woo et al.’s study showed square root of the total error score of 6.29 for Asian subjects and 5.48 for Caucasian subjects. Both of these studies had square root error scores much higher than the 2.72 square root of the total error score found in this study. Another important point of difference is that both Dain et al.’s and Woo et al.’s studies had FM100 scores that showed normality, unlike this study. Because of this, Dain et al. and Woo et al. were able to use square root measures for data analysis, while non-square root measures were used in this study. These factors may have contributed to why significant correlations were not found in our study population. Another likely explanation for lower error scores in this study is that practice effects were accounted for in our study, allowing the observation of stable, plateaued scores.

Normative data proposed by Verriest et al. described a mean square root of the total error score of 6.47 ± 2.42 OD; 6.35 ± 2.39 OS for subjects between the ages of 20–29 years.[14] Kinnear and Sahraie listed a mean square root of the total error score of 6.8 for subjects at age 22 (the 22 year old group being the closest in age as compared to our study’s age average of 25.1).[15] One possibility for the discrepancy between the scores in our study and the norms determined by previous studies is the learning effect as well as the population subtype. Because recruitment for this study occurred from the optometry college at the University, some subjects had at least some familiarity with the FM100; however, no subject reported completing an entire FM100 test previously. Nevertheless, it is possible that scores may have been lower than subjects recruited from the general population. Another possible explanation for the difference in square root of the total error scores is that only 11 subjects were used in the Kinnear and Sahraie study to compute age norms for the 22-year-old group, and only 29 subjects were used for Verriest et al.’s study for the 20–29 year old group. Mahon et al. found an average FM100 of 37.4 in a sample of 126 subjects (mean age 32.2 ± 11 years).[16] Not only is the average age of the participants higher than those in our study, no training or retesting of the FM100 was conducted. This is the most likely explanation for the discrepancy between the results in Mahon et al.’s study and this one.

Theoretically, it may also be that the FM100 test is not the most quantitative, most sensitive test of color discrimination that could be used to investigate a correlation between MPOD and color discrimination. A candidate measure would be the anomaloscope, but subjects had an anomaloscope range of only 12 units as compared to the FM100 test range of 32 units. No other candidate measure is obvious, but one could arise in the future perhaps based on genetic testing.

Another important factor that could explain the discrepancy between the lack of correlation seen in on our study between MPOD and hue discrimination and the positive correlation found in previous studies is the retinal area being used to discriminate FM100 test caps. Studies by Werner et al and Moreland and Westland both established that macular pigment affects color perception in the central foveal region.[9, 29] Macular pigment is most dense at the foveola and its spatial distribution is well described by a Gaussian function with a standard deviation of approximately 1 degree of eccentricity.[25] Thus, by 2 degrees eccentric from the foveola, the MPOD is very low. When testing hue discrimination with the FM100 test, subjects often compare 2–3 caps at a time which would extend beyond the foveola, potentially muting the effects of macular pigment on hue discrimination.

While our sample size of 30 subjects gave enough power to detect a Pearson correlation coefficient of 0.5 or greater in magnitude, a Pearson correlation coefficient of -0.15 was observed, giving a power of 0.12. In order to achieve 0.80 power with a 0.15 correlation an additional ~300 subjects would have had to be recruited. However, even if such a large number of subjects were recruited and a correlation of 0.15 was found to be statistically significant, such a low correlation would not be clinically significant.

### MPOD vs. iris color, iris reflectance, and ethnicity

A strong relationship between MPOD levels and iris color was found, as seen in other studies. The lowest MPOD scores were seen with subjects with hazel eyes, followed by blue-eyed subjects, and brown-eyed subjects were found to have the highest MPOD levels. There was also a strong relation found between MPOD levels and ethnicity—Caucasian subjects were shown to have significantly lower average MPOD levels than Asian subjects. This agrees with the presumptions made in Dain et al.’s and Woo et al.’s studies previously. Another study found that African Americans had significantly higher MPOD than Caucasian subjects.[7] However, no correlation was found between MPOD levels and iris reflectance. Iris reflectance had a high amount of variability by iris color and this likely contributed to the lack of correlation.

In Woo et al.’s study, Asian subjects had poorer blue-yellow discrimination based on FM100 partial errors scores, and it was assumed to be due to the higher amounts of macular pigment (although MPOD was not directly measured) compared to Caucasian subjects.[5] In Dain et al.’s study, subjects were categorized into three groups, based on both ethnicity and iris pigmentation. No significant difference between FM100 scores was found, although results were not separated into partial red-green and partial blue-yellow error scores, and it was concluded that macular pigment does play a role in color discrimination ability.[4] In this study, Asian subjects had a much higher proportion of brown irides and a corresponding lower iris reflectance compared to Caucasian subjects.

The relationship between iris color and MPOD may appear counterintuitive, specifically that hazel-eyed subjects would have lower MPOD values than blue-eyed subjects. The relationship may be clouded by subject ethnicity—especially since subjects were grouped into two major categories of “Caucasian” and “Asian”, as opposed to more specific groupings. To our knowledge, this is the first study that has separated hazel- and blue-eyed subjects into different groups for statistical testing. Hammond and Avery’s study showed that subjects with blue or gray irises (mean MPOD of 0.19) had lower MPOD results than green- or hazel-eyed subjects (mean MPOD of 0.23) or brown- or black-eyed subjects (mean MPOD of 0.24).[6] However, statistical testing only compared “light irises” to “dark irises”, where blue, green or gray irises were grouped together in the “light” category, and hazel, brown or black irises were grouped into the “dark” category. Murray et al. also investigated this relationship and found a significant difference in MPOD between “dark-eyed” (brown-eyed) subjects and “light-eyed” (blue-, grey-, green-eyed) subjects, with mean MPOD values of 0.457 and 0.353 respectively, but again did not differentiate between blue and green iris color.[2] Unfortunately, sample size is a limiting factor in this study for the comparison of the relationship between hazel versus blue eyes and MPOD. Further studies may make the relationship between iris color and macular pigment more clear. Further studies would have to be done with individuals of the same ethnicity but different iris color to determine whether ethnicity or color itself is causing this relationship.

The physiological association of macular pigment and iris color has been proposed by Hammond to be due to either the evolution of a shared tendency to accumulate melanin (iris color) and carotenoids (macular pigment) due to similar environmental factors (e.g. light and oxygen), or the possibility that macular pigment may be depleted because of the tendency for eyes with lighter irises to transmit more light than darker eyes–leading to increased oxidative stress.[30] Thus, iris color and macular pigment could be surrogate measures for each other, which is how Woo et al. and Dain et al. approached the issue.

### MPOD and pupil size

Dain et al. pointed out that Woo et al. did not account for pupil size as an explanation of their findings, and in their own study found that smaller pupil size (which results in lower retinal illuminance) led to poorer FM100 results. This implied that iris color itself may not have correlated with poorer FM100 scores, and instead pupil size played a role. This was not surprising, but did call into question the validity of the Woo et al. results since pupil size was not discussed in their paper. Pupil size (which is a factor to determine retinal illumination) was therefore a confounding variable. While pupil size was measured under standardized conditions, it was not controlled in the sense that all subjects had the same pupil size through an artificial pupil. However, similar to Dain et al. we had restricted age range of subjects, which would ensure more similar ocular media transmission compared to Woo et al.’s study. Additionally, we specifically checked for a statistical correlation between pupil size and MPOD and did not find one.

### Study limitations

Sample size was a limiting factor of this study, especially in regards to the relationship between blue versus hazel iris color and MPOD levels. Future, larger studies with subjects from the same ethnicity but who have differences in iris color would help make this relationship clearer. Most prior studies that have investigated iris color have used a simple scale as used in this study.[31] However, one study proposed a nine-grade scale for iris color and using such a scale coupled with a larger sample size may allow further clarification of associations between iris color and MPOD.[31] Additionally, perhaps our iris reflectance measure was too coarse. Two measurements per quadrant for a total of eight might better capture local iris pigmentation variations and provide a finer metric. A pupillary miotic could also be considered, as this would allow a broader extent of anterior iris surface areas to be visualized and measured. As mentioned previously, another limitation is that the FM100 may not be the most sensitive measure of color vision possible. Anomaloscope testing with larger sample sizes (in order to get a wide range of results) could be used as a more quantitative measure, or perhaps genetic testing in the future. We have discussed the effect of macular pigment likely being more pronounced in the central foveal region; theoretically using filters of differing densities that simulate the macular pigment action spectrum may have elucidated more of a significant difference in FM100 scores, making the issue of central versus extrafoveal viewing less of a factor.

In summary, it is likely that no strong correlation exists between macular pigment optical density and extrafoveal hue discrimination, as determined by the FM100 Hue test. It is possible that in a very large sample size, a significant correlation could be found, but this is unlikely to be clinically significant. Most likely, MPOD only has significant effects on central foveal color discrimination. It is also likely that Woo’s and Dain’s study found associations between MPOD and FM100 Hue scores, where Davison’s didn’t, because of the lack of actual macular pigment density measurement, retinal illumination, and possibly the lack of the determination of the plateau of hue discrimination scores. Ultimately, our results agree with Davison et al. and explain some of the possible associations found by Woo et al. and Dain et al. Clinically, these findings indicate that practitioners do not need to be concerned about MPOD supplementation affecting a patient’s color vision or the need to stratify FM100 normative scores by MPOD level.

## Supporting information

S1 Table Full raw data collected in the study. (PDF)
