# Supplementary material for: Associations between macular pigment, iris color and reflectance, ethnicity, and color vision: An observational study
Source: PLoS One. 2019 Aug 8;14(8):e0220940. doi: 10.1371/journal.pone.0220940 (PMC6687157; doi:10.1371/journal.pone.0220940)
Supplement: S1 Table — (PDF) [file pone.0220940.s001.pdf]

Gender
0-Male
1-Female

Ethnicity
0-Asian
1-Caucasian
2-Hispanic

Eye Cho
0- OD
1- OS

V1- Visit 1
V2- Visit 2

IrisColor
0 = blue
1 = hazel
2 = brown

ID	Age	Gender	Ethnicity	DistVA	NearVA	EyeCho	MPOD	MPOD2	MPOD3	MPODA	TESTV1	PES1TV1	PES2TV	TESTV1sqr	PES1TV1sq	PES2TV1sq	TESV1	PES1V1	PES2V1	TESV1sqrt	PES1V1sqrt	PES2V1sqrt
1	26.94	0	0	-0.06	-0.1	0	0.41	0.46	0.41	0.43	0	0	0	0.00	0.00	0.00	0	0	0	0.00	0.00	0.00
2	23.88	1	1	-0.02	0	0	0.5	0.36	0.5	0.45	32	20	12	5.66	4.47	3.46	20	4	16	4.47	2.00	4.00
3	24.51	1	0	-0.08	-0.04	1	0.55	0.55	0.6	0.57	24	16	8	4.90	4.00	2.83	20	4	16	4.47	2.00	4.00
4	28.41	1	0	-0.06	-0.04	1	0.46	0.41	0.41	0.43	12	2	10	3.46	1.41	3.16	0	0	0	0.00	0.00	0.00
5	24.39	0	1	-0.14	-0.2	0	0.26	0.22	0.37	0.28	0	0	0	0.00	0.00	0.00	0	0	0	0.00	0.00	0.00
6	24.02	1	0	-0.1	-0.06	0	0.55	0.46	0.65	0.55	16	0	16	4.00	0.00	4.00	12	4	8	3.46	2.00	2.83
7	26.08	0	0	-0.12	-0.04	0	0.7	0.55	0.55	0.60	12	0	12	3.46	0.00	3.46	8	0	8	2.83	0.00	2.83
8	27.77	1	2	-0.06	0.12	1	0.5	0.5	0.46	0.49	4	0	4	2.00	0.00	2.00	12	6	6	3.46	2.45	2.45
9	19.34	1	1	-0.08	-0.08	0	0.36	0.22	0.26	0.28	8	8	0	2.83	2.83	0.00	0	0	0	0.00	0.00	0.00
10	26.21	0	1	-0.16	-0.08	0	0.22	0.17	0.17	0.19	28	12	16	5.29	3.46	4.00	24	12	12	4.90	3.46	3.46
11	22.21	0	0	0	0	1	0.36	0.41	0.36	0.38	20	8	12	4.47	2.83	3.46	8	4	4	2.83	2.00	2.00
12	25.74	1	0	-0.08	-0.08	0	0.46	0.41	0.44	0.44	12	8	4	3.46	2.83	2.00	4	0	4	2.00	0.00	2.00
13	23.84	0	1	-0.1	-0.1	0	0.26	0.31	0.31	0.29	0	0	0	0.00	0.00	0.00	4	0	4	2.00	0.00	2.00
14	28.69	0	1	-0.02	-0.04	1	0.55	0.55	0.55	0.55	12	0	12	3.46	0.00	3.46	8	0	8	2.83	0.00	2.83
15	22.70	1	1	-0.02	-0.04	0	0.41	0.41	0.36	0.39	0	0	0	0.00	0.00	0.00	0	0	0	0.00	0.00	0.00
16	24.13	0	1	-0.1	-0.04	1	0.26	0.26	0.12	0.21	16	4	12	4.00	2.00	3.46	20	2	18	4.47	1.41	4.24
17	24.30	0	1	-0.1	-0.14	0	0.41	0.46	0.41	0.43	12	8	4	3.46	2.83	2.00	12	4	8	3.46	2.00	2.83
18	30.90	0	1	-0.04	-0.06	0	0.36	0.41	0.31	0.36	0	0	0	0.00	0.00	0.00	0	0	0	0.00	0.00	0.00
19	22.15	1	1	-0.04	-0.04	1	0.22	0.17	0.17	0.19	12	12	0	3.46	3.46	0.00	8	0	8	2.83	0.00	2.83
20	24.41	0	0	-0.14	-0.04	0	0.41	0.46	0.46	0.44	20	4	16	4.47	2.00	4.00	20	0	20	4.47	0.00	4.47
21	25.03	1	1	0	-0.02	1	0.65	0.7	0.55	0.63	0	0	0	0.00	0.00	0.00	4	0	4	2.00	0.00	2.00
22	22.55	1	1	-0.02	0	0	0.41	0.31	0.41	0.38	12	4	8	3.46	2.00	2.83	8	8	0	2.83	2.83	0.00
23	22.18	1	1	-0.1	-0.1	0	0.36	0.36	0.26	0.33	24	8	16	4.90	2.83	4.00	24	4	20	4.90	2.00	4.47
24	28.38	1	1	-0.08	-0.02	1	0.22	0.17	0.22	0.20	8	0	8	2.83	0.00	2.83	8	4	4	2.83	2.00	2.00
25	24.97	1	1	0.04	-0.04	1	0.46	0.46	0.5	0.47	24	8	16	4.90	2.83	4.00	20	12	8	4.47	3.46	2.83
26	24.02	0	1	-0.1	0.02	1	0.17	0.17	0.17	0.17	20	0	20	4.47	0.00	4.47	32	8	24	5.66	2.83	4.90
27	25.23	1	1	-0.02	-0.02	0	0.36	0.36	0.26	0.33	32	16	16	5.66	4.00	4.00	32	16	16	5.66	4.00	4.00
28	27.32	0	1	-0.04	0	0	0.36	0.41	0.46	0.41	0	0	0	0.00	0.00	0.00	0	0	0	0.00	0.00	0.00
29	28.48	1	1	-0.12	0.02	1	0.26	0.26	0.41	0.31	8	0	8	2.83	0.00	2.83	8	4	4	2.83	2.00	2.00
30	25.44	1	1	-0.12	-0.14	0	0.22	0.17	0.22	0.20	12	0	12	3.46	0.00	3.46	4	0	4	2.00	0.00	2.00

ID	TESV2	PES1V2	PES2V2	TESV2sqrt	PES1V2sq	PES2V2sq	MPOD1V2	MPOD2V2	MPODAveV	RGMidpo	RGRange	BYMidpo	IrisReflec	PupilSi	IrisColor
1	0	0	0	0.00	0.00	0.00	0.36	0.46	0.41	42	2	77.25	19.40	5.5	2
2	36	16	20	6.00	4.00	4.47	0.36	0.5	0.43	42.25	7.5	56.58	14.72	6	0
3	24	8	16	4.90	2.83	4.00	0.5	0.6	0.55	46.75	3.5	49.67	0.54	5	2
4	0	0	0	0.00	0.00	0.00	0.46	0.41	0.435	41.5	7	74.08	9.46	5.5	2
5	0	0	0	0.00	0.00	0.00	0.26	0.26	0.26	49	1	80.42	17.96	5.5	2
6	4	0	4	2.00	0.00	2.00	0.5	0.55	0.525	43.5	7	66.58	21.27	5.5	2
7	4	0	4	2.00	0.00	2.00	0.65	0.65	0.65	42.5	13	70.67	3.90	4.5	2
8	4	0	4	2.00	0.00	2.00	0.6	0.46	0.53	46.5	9	66.33	2.04	3.5	2
9	0	0	0	0.00	0.00	0.00	0.31	0.26	0.285	39.5	5	52.00	23.99	6.5	1
10	16	4	12	4.00	2.00	3.46	0.21	0.17	0.19	40.75	7.5	67.08	26.24	5.5	0
11	20	4	16	4.47	2.00	4.00	0.36	0.41	0.385	41	4	47.92	1.59	5	2
12	0	0	0	0.00	0.00	0.00	0.46	0.5	0.48	41	4	29.50	0.98	4.5	2
13	0	0	0	0.00	0.00	0.00	0.22	0.26	0.24	45	4	71.75	15.83	5.5	0
14	24	8	16	4.90	2.83	4.00	0.5	0.6	0.55	44	4	61.00	25.81	3	0
15	0	0	0	0.00	0.00	0.00	0.41	0.36	0.385	42	4	75.83	21.67	8	0
16	24	4	20	4.90	2.00	4.47	0.17	0.22	0.195	41.5	7	61.92	8.07	8	1
17	8	0	8	2.83	0.00	2.83	0.46	0.5	0.48	39.25	4.5	55.08	4.72	3	1
18	0	0	0	0.00	0.00	0.00	0.41	0.41	0.41	42	4	64.58	3.83	5	1
19	16	8	8	4.00	2.83	2.83	0.26	0.22	0.24	43.5	5	57.00	15.14	5.5	1
20	12	8	4	3.46	2.83	2.00	0.46	0.46	0.46	41.75	3.5	67.92	0.89	6.5	2
21	12	4	8	3.46	2.00	2.83	0.6	0.6	0.6	42.25	8.5	61.50	6.18	4.5	1
22	4	0	4	2.00	0.00	2.00	0.26	0.26	0.26	43	4	54.58	10.35	5.5	1
23	24	8	16	4.90	2.83	4.00	0.31	0.26	0.285	43	8	50.08	8.69	7	0
24	4	4	0	2.00	2.00	0.00	0.22	0.26	0.24	40.5	3	43.75	19.37	5	0
25	32	4	28	5.66	2.00	5.29	0.5	0.46	0.48	42	6	53.83	21.91	5	0
26	20	8	12	4.47	2.83	3.46	0.17	0.17	0.17	41.25	10.5	56.58	16.23	5.5	0
27	16	4	12	4.00	2.00	3.46	0.36	0.31	0.335	41.5	13	59.67	3.23	7.5	0
28	4	4	0	2.00	2.00	0.00	0.41	0.41	0.41	41.5	5	42.83	3.33	7.5	0
29	0	0	0	0.00	0.00	0.00	0.41	0.31	0.36	44.5	7	60.83	1.65	5.5	1
30	8	4	4	2.83	2.00	2.00	0.12	0.22	0.17	42.5	7	49.58	1.27	4.5	1
